# Effectiveness of repetitive transcranial magnetic stimulation against poststroke urinary incontinence: a study protocol for a randomized controlled trial

**DOI:** 10.1186/s13063-022-06535-y

**Published:** 2022-08-13

**Authors:** Wei Jiang, Wen Tang, Yunling Song, Yali Feng, Yuesan Zhou, Lang Li, Botao Tan

**Affiliations:** Department of Rehabilitation Medicine, The Second Affiliated Hospital of Chongqing Medical University, 74 Linjiang Road, Chongqing, 40010 China

**Keywords:** rTMS, Stroke, Urinary incontinence, Clinical trial, Bladder

## Abstract

**Background and purpose:**

Poststroke urinary incontinence (PSI) is prevalent in stroke survivors, and high-quality evidence is required to guide clinical practice. Previous studies have demonstrated the curative effect of repetitive transcranial magnetic stimulation (rTMS) for urinary incontinence in individuals with multiple sclerosis (MS), Parkinson’s disease (PD), and spinal cord injury (SCI). Here, we describe the protocol for a randomized controlled trial to evaluate the efficacy and safety of low-frequency rTMS on the contralesional primary motor cortex (M1) for the treatment of PSI.

**Methods and analysis:**

In this single-centre randomized controlled trial for poststroke urinary incontinence, a total of 140 eligible patients will be randomly allocated into two groups. The rTMS group (*n* = 70) will receive low-frequency rTMS at the M1 along with routine medical care, while the control group will receive sham rTMS along with routine medical care. All participants will undergo 20 treatment sessions, five times a week for 4 weeks. The primary outcome measures will be the changes in the urodynamic test at baseline versus 4 weeks after intervention. The secondary outcomes include the International Consultation on Incontinence Questionnaire Urinary Incontinence Short Form (ICIQ-UI SF), Overactive Bladder Symptom Score (OABSS), and pelvic floor muscle function.

**Ethics and dissemination:**

The Institutional Review Board and Hospital Research Ethics Committee of the Second Affiliated Hospital of Chongqing Medical University approved this trial, and the approval number is No. 2020-153. All methods will be carried out in accordance with the principles of the Declaration of Helsinki and relevant ethical guidelines covering informed consent, confidentiality, and data storage. After the study had been thoroughly described to the participants by a physician, all participants will provide written informed consent indicating their willingness to participate. The results will be disseminated to most of the population, including participants, researchers, healthcare providers, and sponsors.

**Trial registration:**

URL: https://www.chictr.org.cn; Unique identifier: ChiCTR2100042688. Date of Registration: 2021-01-26.

**Supplementary Information:**

The online version contains supplementary material available at 10.1186/s13063-022-06535-y.

## Introduction

Poststroke urinary incontinence (PSI) is a common complication that presents as involuntary loss of urine. PSI has been reported to affect 40~ 60% of patients admitted to the hospital after a stroke, with up to 25% still having problems when discharged from the hospital and 15% remaining incontinent after one year [1–3]. Recent studies have shown that up to 79% of stroke survivors develop urinary incontinence [4]. Typical symptoms of poststroke incontinence include urinary urgency immediately followed by involuntary leakage of urine, which causes skin dermatitis and urinary tract infections [5, 6]. It has also been reported to be associated with psychological problems such as low self-esteem and anxiety [7]. Moreover, the presence of urinary incontinence has been recognized as a marker of stroke severity and a higher mortality rate than those without urinary incontinence [8]. Accordingly, the management of PSI is of critical importance and should be the aim of all stroke health professionals.

In clinical practice, the management of PSI mainly relies on nurses [6]. A thorough assessment is required to delineate the type and severity of incontinence so that treatment can be tailored to meet the patient’s needs. In addition to the International Consultation on Incontinence Questionnaire Urinary Incontinence Short Form (ICIQ-UI SF) and urinary diary, subjective assessments such as pelvic floor examination and urodynamic tests are also recommended [4, 9–12]. Previous studies demonstrated that detrusor overactivity (DO) takes over the major urodynamic patterns of PSI [10, 11]. Therefore, strategies targeting detrusor overactivity are an important aspect of rehabilitation care. However, compliance is always a problem for patients with catheters or external collecting devices [13]. Oral medication of detrusor overactivity is associated with several complications such as cognitive damage and dry mouth [3, 14]. The effectiveness of behaviour training, acupuncture, and pelvic floor muscle exercises remains unclear [1, 7, 15–17]. Therefore, further investigation is needed to establish an effective therapy for PSI patients with DO.

The bladder is controlled by a supratentorial network via a long-loop reflex pathway from the motor cortex to the sacral spinal cord [18, 19]. Brain damage, such as stroke, could cause urinary dysfunction. Emerging research on the central pathophysiologic mechanisms of incontinence with bladder overactivity suggests that the brain is a potential target for therapeutic interventions [19]. Repetitive transcranial magnetic stimulation (rTMS) is a newly developed noninvasive brain stimulation method for the treatment of neurological disorders. It can modulate cortical excitability and induce long-lasting neuroplastic changes when it is applied over the cortical areas corresponding to the pelvic region [20, 21]. Studies have demonstrated that high frequency (≥ 5 Hz) rTMS on the primary motor cortex (M1) can improve detrusor contraction in patients with multiple sclerosis (MS) and incomplete spinal cord injury, while low frequency (≤ 1 Hz) rTMS on M1 seems to inhibit bladder activity in subjects with Parkinson’s disease [21–23]. However, the clinical effectiveness of rTMS on poststroke urinary incontinence lacks evidence and is worthy of investigation in a well-designed study. To our knowledge, this is the first randomized controlled trial (RCT) to explore the effect of rTMS on reducing detrusor overactivity and relieving the symptoms of incontinence in stroke patients. Our primary aim was to investigate the changes in urodynamic tests after low-frequency rTMS on M1. We also plan to observe the effectiveness of rTMS on ICIQ-UI SF and pelvic floor muscle activity.

## Methods

This is a single-centre, randomized double-blind, placebo-controlled superiority trial. Patients will be randomized 1:1 to receive either rTMS or sham rTMS therapy. The sham rTMS was believed to be an inactive treatment, so it is a plausible comparator. The authors declare that all supporting data will be available online or by email contact with the corresponding author.

### Participants

Stroke patients with urinary incontinence will be recruited by flyers posted in the rehabilitation department and rehabilitation medicine outpatient clinic waiting areas. Each participant will be initially interviewed and evaluated by an experienced rehabilitation physician, and the evaluation will be conducted by a well-trained nurse on the research team.

The inclusion criteria were as follows [16, 24]: (1) onset of stroke within 1 and 12 months of enrolment, (2) suffered from urinary incontinence after stroke, (3) aged over 30 years of any sex, (4) no special medications administered for urinary incontinence, and (5) the ability to understand and agree to the trial procedures and to sign an informed consent form (online supplementary Additional file 1) in accordance with the national legislation.

The exclusion criteria were as follows: (1) any urinary dysfunction before stroke, (2) bilateral lesion in the brain, (3) urinary tract infection, (4) the presence of an unstable medical condition or an uncontrolled known systemic disease, (5) contraindications to rTMS (i.e., seizure, cardiac pacemaker, or ear nest implants), and (6) refusal to continue the intervention.

### Randomization and masking

The study will be conducted at the Second Affiliated Hospital of Chongqing Medical University. Participants will be randomly allocated to receive low frequency (LF) rTMS or sham rTMS over the contralesional primary motor cortex (M1). The randomized number and sequence will be generated through SPSS Version 19.0 by an independent statistician who will not take part in assessments and execution in this trial. An outside research assistant will allocate these numbers to participants according to the order of inclusion. After that, the therapist and all eligible participants will be informed of the results of group allocation by an independent clinician via short message.

Given the physicians and therapists will administer the rTMS protocol, they cannot be blinded. However, both the researchers (i.e., outcome assessors and statistical analysts) and participants will be blinded to the group assignment, and the blindness will never be broken prior to the completion of the study unless adverse events occur. Additionally, the randomized sequence of allocation will be replaced by unrelated codes, code I and code II. In this study, code I is defined as the rTMS group, and code II is defined as the sham rTMS group. Each parameter of randomization will be kept in a special sealed opaque envelope as previously reported [25].

### Interventions

**Fig. 1:**
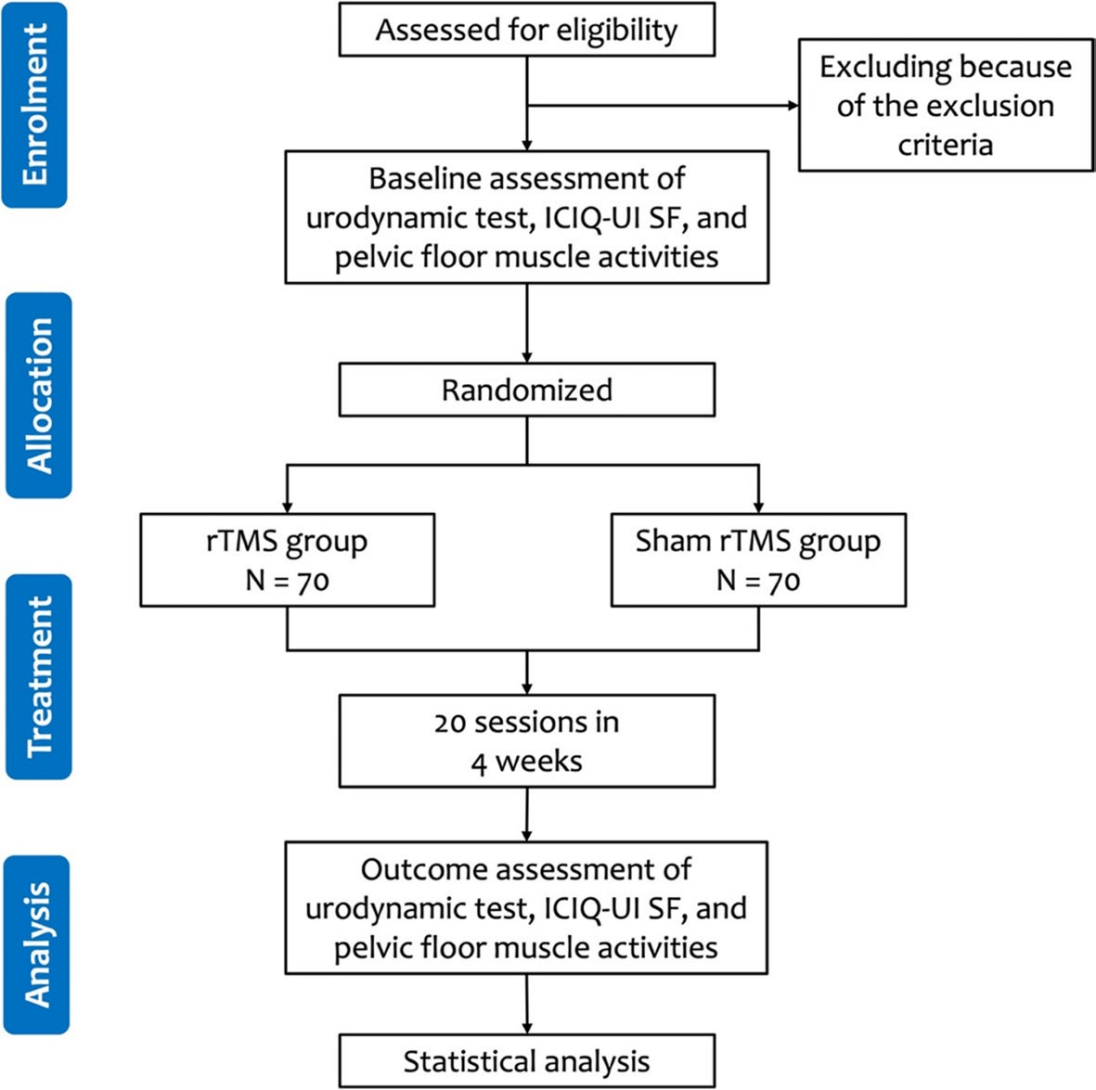
Proposed participant flow

This study is designed according to the CONSORT 2010 statement, and the protocol follows the Standard Protocol Items: Recommendations for Interventional Trials (SPIRIT) guidelines and fulfils the SPIRIT checklist (online supplementary Additional file 2) [26, 27]. The study flowchart is shown in Fig. 1. All participants who fulfil the inclusion criteria will be assessed. Specifically, a detailed medical history will be taken, and information on patients’ age, sex, weight, and height will also be collected.

**Fig. 2:**
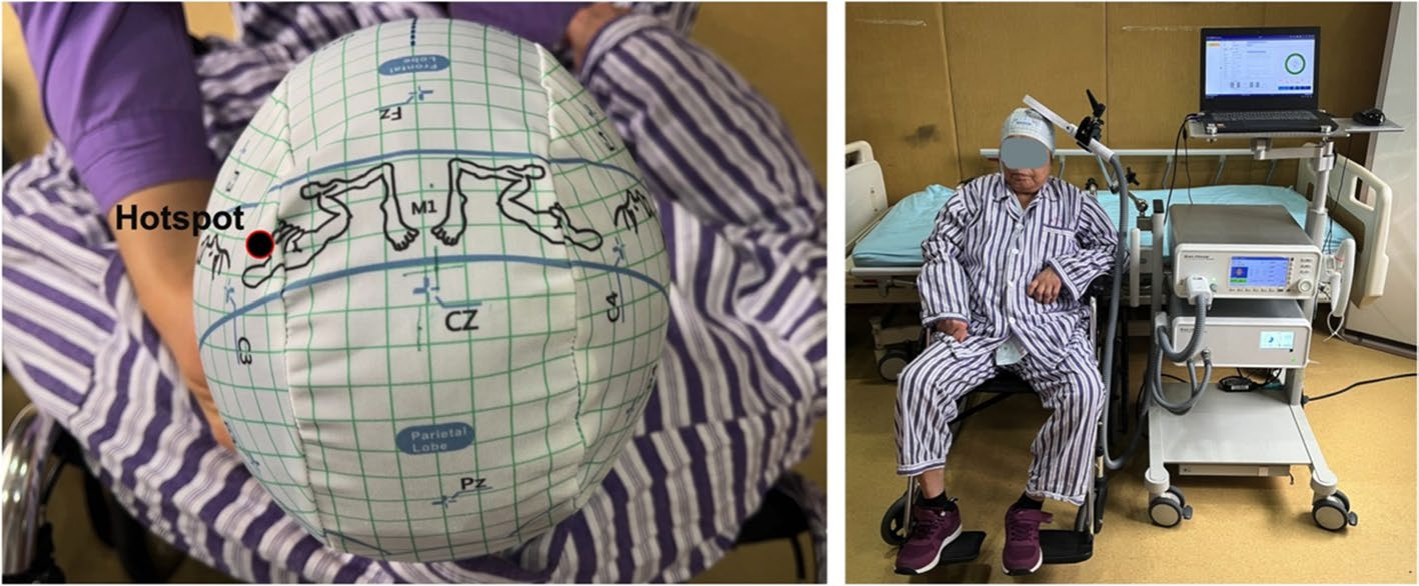
Locations of the hotspots for rTMS. This is a left hemiplegia patient with an ischaemic stroke on the right side of the cortex. Black dots with red circles show the hotspot. The patient will sit on the chair when they accept the intervention

As studies have demonstrated that LF (≤ 1 Hz) rTMS on M1 seems to inhibit bladder activity in subjects with Parkinson’s disease [21, 22], we hypothesized that LF rTMS would be effective in relieving detrusor activity, enlarging the bladder and hence relieving the symptoms of urinary incontinence in poststroke survivors. Therefore, participants in the rTMS group will receive LF rTMS over the hotspot of the contralesional primary motor cortex (M1) using a figure-of-8 coil for a 4-week period [21, 28] (Yingchi Technology Co. Ltd) (Fig. 2). A low frequency of 1 Hz with a stimulus intensity of 80% of resting motor threshold (rMT) of the nonparetic hand for a total of 1200 pulses will be used in each session. All rTMS treatments will be conducted by a well-trained physiotherapist. Sham rTMS will be conducted using a sham 8 coil, which produces a similar sound without stimulation.

All patients will receive a regular course of rehabilitation therapy [29], nursing management of the bladder [30, 31] and necessary medications (to control blood pressure, blood sugar and cholesterol, but not for bladder dysfunction) during the 4-week period of intervention or following the administration of sham stimulation.

### Outcome assessment

Clinical assessments of the participants will be performed at baseline and 4 weeks post-intervention. Urodynamic testing is the gold standard for the evaluation of lower urinary tract dysfunction [32]. Therefore, we will employ urodynamic testing to evaluate bladder function, and bladder capacity (one of the urodynamic testing outcome indicators) will be recognized as the primary outcome. Other outcomes, such as detrusor pressure and residual volume, will also be obtained from urodynamic testing. The multichannel urodynamic test will be conducted according to standards recommended by the International Continence Society [33]. This exam will be conducted using a multichannel urodynamic test system (Nidoo 970A+, China), with a usual fill rate of 30 mL/min. Urodynamic data to be collected includes the presence of involuntary detrusor contraction, bladder compliance, sensation of bladder filling, any leakage, bladder capacity, detrusor pressure at maximum flow, maximum flow rate, and volume of postvoid residual urine.

The self-reported questionnaire of the ICIQ-UI SF will be used to assess the prevalence, frequency, perceived cause of UI, and influence on everyday life. The Chinese version of the five ICIQ modules can be easily completed with a Cronbach's alpha coefficient of 0.71–0.96 [34]. The total ICIQ-UI SF score ranges from 0 to 21 points, with the severity being classified according to the total score: 0–7 points for mild UI, 8–12 points for moderate UI, and 13–21 points for severe UI [35]. The Overactive Bladder Symptom Score (OABSS) will also be used to indicate symptom severity, with the total score varying from 0 to 15 [36, 37].

The electromyography activity by means of surface EMG (sEMG) recordings will be employed to assess the pelvic floor muscle (PFM) activities [38, 39]. In brief, participants will be asked to empty their bladder and rectum and will be instructed on how to contract the PFMs. A probe will be inserted into the correct place of the vaginal (for females) or rectal (for males) region, and the PFM activity at rest will be recorded for 30 s, followed by two 5-s maximum voluntary contractions (MVCs) with a break of 15 s in between. The sEMG activity onset threshold will be considered the mean plus two standard deviations of the filtered and rectified EMG during the 30 s rest according to Baur et al. [40]. The sEMG outcomes will be calculated as root mean square (RMS) values within the respective time intervals and averaged over 10 s.

All patients will receive assessment at 0 days after enrolment, 14 and 28 days after start of treatment, and 3 months at the end of the trial for follow-up. Scoring data are collected, as described in supplement Statistic Analysis Plan.

All participants will be able to withdraw from the study at any time for any reason. If a participant discontinues or deviates from intervention protocols, the investigator will report the withdrawal and the reasons for the withdrawal and obtain measurements of the time of the last treatment and possible outcomes, such as the urodynamic test outcomes.

### Safety assessment

Although the existing research on rTMS treatment of stroke patients has not found the occurrence of major adverse reactions, the known potential risks, such as epilepsy, will be highly regarded. Other potential reactions, such as fatigue, dizziness, and headache, will be recorded to evaluate the safety of rTMS for poststroke patients with urinary incontinence. The questionnaires will be administered to the participants after every verum or sham rTMS session, and the results will be recorded and analysed. The outcome assessors will judge the severity (mild, moderate, or severe), seriousness, and causality (definitely related, probably related, possibly related, possibly not related, definitely not related to the intervention, or not assessable).

According to the safety guidelines, the appropriate parameters of rTMS will be discussed with each of the physicians and the therapists administering the TMS. All researchers need to have adequate skills to master the management of seizures as well as other medical emergencies. This trial will take place in a general hospital where life-support equipment is available. Meanwhile, the Data Monitoring Committee (DMC) will help to complete interim analyses or stop rules. The composition of the DMC includes both clinicians and statisticians, and it is independent of the sponsors and the research team. The DMC is responsible for safety monitoring and applying appropriate statistical methods. Additionally, it will review accumulating data from an ongoing clinical trial and make recommendations that might impact the future conduct of the trial to ensure that there is no unavoidable increased risk for patients participating in such trials. If there are high numbers of protocol violations/deviations or high numbers of patients who withdraw from the study, the DMC may recommend stopping the trial. Considering the potential adverse reactions and the physiological state of the stroke survivors, we anticipate a 15~20% dropout rate.

If any participants suffer harm from the trial, they will receive medical treatment for the injury or complication, free of charge, as a public patient in the Second Affiliated Hospital of Chongqing Medical University.

### Sample size

We aim to investigate the efficacy of LF rTMS for improving bladder function in PSI individuals. However, the primary outcomes for most previous studies are the pad test and urinary continence questionnaire, including the study previously referenced [41]. In this trial, we chose bladder capacity as one of our primary outcomes. Based on our previous observation (unpublished data), the mean and SD of bladder capacity in PSI patients were approximately 300 and 100 ml, respectively, and a bladder capacity increase of more than 50 ml was thought to be a therapeutic effect [42]. According to those results, a sample size of 63 participants in each group was calculated to sufficiently detect the target effect size (0.5) with a type I error of 5% (*α* = 0.05) and 80% power (*β* = 0.20) by Gpower V.3.1.9.2 software. We will add 10 to 25% more participants to account for potential loss to follow-up, resulting in a final enrolment goal of 140 participants (70 per group).

### Statistical analysis

All statistical analyses will be performed by a statistician from the Clinical Evaluation and Analysis Centre of the Second Affiliated Hospital of Chongqing Medical University using SPSS 19.0 (SPSS, Inc., Chicago, IL), and *P* < 0.05 will be considered statistically significant (two tail). If participants drop out during the intervention period, they will not be excluded due to the principle of intention to treat (ITT), and the missing data will be replaced by the last observation.

Continuous variables with a normal distribution will be expressed as the mean and standard deviation or median interquartile range. First, the comparison of the baseline data between the two groups will be performed using Student’s *t* test, chi-squared test, or nonparametric test according to the type and distribution of variables. To verify the therapeutic efficacy of our intervention, comparisons of all the outcomes that are categorized as continuous variables and shown in mean scores will be included in the analysis by independent sample *t-*test or non-parametric test (Wilcoxon and Mann–Whitney *U* tests) depending on the distribution of the data [16, 25]. If the data are distributed normally and the variances are equal, independent samples *t-*tests will be used for comparisons of the before and after treatment changes between groups, including the mean differences between groups, with 95% CIs. For a detailed statistical analysis plan, please see online supplementary Additional file 3.

Our hypothesis is that real rTMS stimulation will elicit greater changes in balder capacity than sham rTMS stimulation. Therefore, the main analysis is the comparison of changes between groups, measured pre- and post-intervention. If significant differences are found before and after treatment changes between groups, we may conclude the efficacy of rTMS to participants with PSI.

### Patient and public involvement

Patients and the public will not be directly involved in the planning and design of this study. Considering strokes are an incurable chorionic disease, the outcome measures to be evaluated in this study will potentially be influenced by the sociodemographic characteristics and preferences of the patients. Therefore, we will use the urodynamic test as our primary outcome assessment. After finishing the study, the results will be disseminated in peer-reviewed journals and at academic conferences.

### Modification of the protocol

Any modifications to the protocol, including changes in the objectives, design, patient population, sample sizes, and study procedures, will require a formal application from the Institutional Review Board (the Board) and Hospital Research Ethics Committee (the Committee) of the Second Affiliated Hospital of Chongqing Medical University. Based on the data from the safety assessment, if the research is changed to eliminate an apparent immediate hazard(s) to the subject, the investigator will promptly notify the Board and the Committee of the change(s). The Board and the Committee review at the next convened meeting to determine if the change(s) instituted were consistent with the subject's continued welfare.

### Confidentiality and monitoring

Only researchers who participated in this study can access the data. All participants of the study will be given an identification number throughout the trial to assure confidentiality. The data of all participants will be recorded into the designed case report forms (CRFs), and each CRF will be checked twice to ensure accuracy and completion. Meanwhile, all related documents will be coded by specific identification codes and stored in a secure manner in the Department of Rehabilitation Medicine of the Second Affiliated Hospital of Chongqing Medical University. The paper version of the recorded data will be entered into the Excel form for management by two physicians with double-checking. The electronic data will be stored on a double password-protected computer. Only the primary investigator and the statistician will own the password. According to the rules of medical file preservation and the principle of Good Clinical Practice (GCP), these materials will be preserved for at least 5 years after the completion of this study.

For quality control, two clinical physicians who are not directly involved in the research team will conduct regular on-site monitoring visits to ensure that all the contents of the research protocol are strictly observed and check the original data to ensure they are consistent with the contents on the CRF. They are also responsible for long-term patient communication and health education. The above measures can improve patient compliance and reduce the failure rate of follow-up.

## Discussion

Although over half of the stroke patients admitted to the hospital have urinary incontinence, there is very little evidence for clinical practice from stroke-specific studies in managing PSI [1]. Therefore, high-quality evidence from RCTs for poststroke urinary incontinence is still needed. To our knowledge, this is the first randomized trial comparing rTMS and sham rTMS for neurogenic bladder dysfunction in stroke patients. We aim to evaluate the improvement of bladder function in PSI patients with LF rTMS of the contralesional M1.

In recent years, accumulating studies have shown that rTMS produces remarkable clinical effects in patients with various neurological and psychiatric disorders [28, 43]. The updated analysis recommended level A evidence for rTMS on neuropathic pain, depression and hand motor recovery in the post-acute stage of stroke. It has been reported that LF rTMS is able to reverse low urinary tract disturbances in patients with PD [22], enhancing bladder capacity and the first sensation of bladder filling. However, high-frequency rTMS applied over the motor cortex ameliorated the voiding phase of the micturition cycle and thus might be useful for increasing detrusor contraction in MS patients [23]. Therefore, a protocol of LF rTMS (1 Hz) over the contralesional M1 was selected in the current study. Interestingly, this protocol is very similar to those studies on rTMS in improving upper extremity function in the post-acute stage of stroke [44, 45]. However, unlike recent study protocols [12, 16, 24], our primary outcome will rely on urodynamic testing, which reduces the bias of subjective assessment from the patients and the assessors. However, the ICIQ-UI SF and OABSS will also be used as subjective measures to investigate UI symptoms and QoL. The status of pelvic floor muscles is closely related to urinary incontinence symptoms, and its electrical activity is widely used to reflect objective muscle contraction abilities [12, 40]. Thus, both subjective and objective data regarding bladder function and QoL will be collected using self-reported questionnaires.

In conclusion, we described a protocol for a randomized clinical study to investigate the efficacy and safety of primary motor cortex (M1) LF rTMS on poststroke urinary incontinence. The results of this RCT will provide scientific evidence for the rehabilitation management of stroke patients with bladder dysfunction.

## Supplementary Information

**Additional file 1.** Informed consent form.

**Additional file 2.** SPIRIT 2013 Checklist.

**Additional file 3.** Statistical Analysis Plan.

## Data Availability

Not applicable.

## Abbreviations

RCT: Randomized control trial
rTMS: Repetitive transcranial magnetic stimulation
LF: Low frequency
M1: Primary motor cortex
MS: Multiple sclerosis
PD: Parkinson’s disease
ICIQ-UI SF: International Consultation on Incontinence Questionnaire Urinary Incontinence Short Form
OABSS: Overactive Bladder Symptom Score
sEMG: Surface electromyography
PFM: Pelvic floor muscles
