# Supplementary material for: Effectiveness of repetitive transcranial magnetic stimulation against poststroke urinary incontinence: a study protocol for a randomized controlled trial
Source: Trials. 2022 Aug 13;23:650. doi: 10.1186/s13063-022-06535-y (PMC9375329; doi:10.1186/s13063-022-06535-y)
Supplement: Supplementary file 1 — Additional file 1. Informed consent form. [file 13063_2022_6535_MOESM1_ESM.docx]

**INFORMED CONSENT FORM**

**Version 2.0 Date 2020.10.1**

**Dear Prospective Participant**

We invite you to participate in the research project of "Low frequency repetitive transcranial magnetic stimulation against post stroke urinary incontinence ". This study will be conducted by the Second Affiliated Hospital of Chongqing Medical University and has been reviewed and approved by the Research Ethics Committee of the Second Affiliated Hospital of Chongqing Medical University.

1. Purpose of the project

Post-stroke urinary incontinence is prevalent in stroke survivors and high-quality evidence is required in guideline clinical practice. Previous studies demonstrated the curative effect of rTMS urinary incontinence on MS, Parkinson’s disease (PD), and spinal cord injury (SCI). We designed the randomized controlled trial for evaluation of the efficacy and safety of low frequency rTMS on the contralesional primary motor cortex(M1) for the treatment of PSI.

2. Explanation of Procedures

All subjects will receive observations and baseline assessment after obtaining informed consent, only who met inclusion/ exclusion criteria will be included in the trial and randomized. Eligible participants are randomized to one of two arms: 1) rTMS treatment (1Hz rTMS on the controlesional motor cortex) plus traditional nursing management; 2) Sham rTMS treatment(only sound will be heard) plus traditional nursing management. They will receive a four-week intervention and complete evaluation of outcome indicators at base line and 4 weeks after the intervention.

3. What other treatment options are available?

Manipulation treatment for post-stroke urinary incontinence, mainly include, 1) pharmacological (such as Tolterodine, Botox injection), and 2) surgical interventions, 3) acupuncture. All these treatments are not definitely effective because it is lack of clinical evidence. And conventional nursing includes the use of supportive devices and behavioral interventions. The nurses will give you suitable management no matter which group you are in.

4. Population who didn't be suitable for joining research

You can't participant this project if you have 1) any urinary dysfunction before stroke, (2) bilateral lesion in the brain, (3) currently urinary tract infected, (4) the presence of an unstable medical condition or an uncontrolled known systemic disease, (5) metal in cranium, intracardiac lines, increased intracranial pressure, heart disease, cardiac pacemaker, medication pump, tricyclic anti-depressants, neuroleptics or family history of epilepsy so that they can’t receive rTMS treatment, 6) refuse to continue of the intervention, and 7) are currently participating in other research relating to your UI.

5.Discomfort and Risks

The biofeedback and repeated transcranial magnetic stimulation involved in this project are all safe and effective treatment methods. However, you may feel some discomfort if you have bad posture, excessive hunger, fatigue, dizzy, mental stress during treatment. To prevent excessive fatigue, you should avoid strenuous exercise before treatment. There may be other possible risks in this procedure, including muscle soreness, fatigue, weakness, and the tingling caused by the current acting on the human body. Generally, the discomfort feel can be relief by adjusting the training intensity and getting appropriate rest.

6. Benefits

By participating in this study, your condition may be improved. In addition to, this research will help you learn more about your disease and also help to choose a more safe and effective treatment method for treating other patients with similar conditions in future.

7. Fees

If you participant this research, you will receive free rehabilitation assessments, free outpatient follow-up and telephone follow-up. Rehabilitation treatments included in this trial are routine clinical treatment methods, thus, the costs incurred are borne by the patients. If the subjects have adverse reactions or serious adverse reactions related to the rehabilitation training of this trial, you can obtain active treatment for the adverse reactions and appropriate reduction of the costs incurred.

8. Confidentially

All information you supply during the research will be held in confidence and unless you specifically indicate your consent, your name will not appear in ang report or publication of the research. Your data will be safely stored in a locked facility and only research staff will have access to this information. Confidentiality will be provided to the fullest extent possible by law.

9. Voluntary Participation

Your participation in the study is completely voluntary and you may choose to stop participating at any time. Your decision will not influence the treatment you may be receiving and nature of the ongoing relationship you may have with the researchers or study staff. If you decide to withdraw from this study, please contact your doctor.

Subject Statement: I have read and understand the explanation provided to me. I have had all my questions answered to my satisfaction, and I voluntarily agree to participate in this study.

I agree□ or reject□ other research uses my research data and biological specimens in addition to this research.

Printed Name of Participant:

Signature of Participant: Date:

Phone of Participant:

Printed Name of Legal representative: (If applicable)

Relationship with subjects:

Signature of Legal representative: Date:

Reason for signing by legal representative:

Printed Name of Witness: (If applicable)

Signature of Witness: Date:

Reason for signing by Witness

Doctor Statement: I have explained the relevant information of the study to the volunteer who participated in this study and provided him / her with an original signed informed consent form. I confirm that I have explained the details of this study to the subject and answered his/her all my questions.

Signature of Doctor: Date:

Phone of Doctor:

**Biomedical Research Ethics Committee， Second Affiliated Hospital of Chongqing Medical University**

**Phone: 023-63693075**
