# Supplementary material for: Effectiveness of repetitive transcranial magnetic stimulation against poststroke urinary incontinence: a study protocol for a randomized controlled trial
Source: Trials. 2022 Aug 13;23:650. doi: 10.1186/s13063-022-06535-y (PMC9375329; doi:10.1186/s13063-022-06535-y)
Supplement: Supplementary file 3 — Additional file 3. Statistical Analysis Plan. [file 13063_2022_6535_MOESM3_ESM.docx]

Statistical Analysis Plan

# SAP Signatures

I give my approval for the attached SAP entitled <Effectiveness of repetitive transcranial magnetic stimulation against poststroke urinary incontinence>. Version 1.0 Date: 20220.12.30

**Statistician**

Name:

Signature:                                                

Date:

**Statistician Reviewer (As applicable)**

Name:

Signature:                                                

Date:

**Principal Investigator**

Name:

Wei Jiang

Signature:                                                

Date:

# Table of Contents

[1 SAP Signatures 1](#_Toc107238570)

[2 Table of Contents 2](#_Toc107238571)

[3 Abbreviations and Definitions 4](#_Toc107238572)

[4 Introduction 5](#_Toc107238573)

[4.1 Preface 5](#_Toc107238574)

[4.2 Scope of the analyses 5](#_Toc107238575)

[5 Study Objectives 5](#_Toc107238576)

[5.1 Study Objectives 5](#_Toc107238577)

[6 Study Methods 5](#_Toc107238578)

[6.1 General Study Design and Plan 5](#_Toc107238579)

[6.2 Inclusion-Exclusion Criteria and General Study Population 6](#_Toc107238580)

[6.3 Randomization and Blinding 6](#_Toc107238581)

[6.4 Study Assessments 6](#_Toc107238582)

[7 Sample Size 7](#_Toc107238583)

[8 General Analysis Considerations 7](#_Toc107238584)

[8.1 Timing of Analyses 7](#_Toc107238585)

[8.2 Analysis Populations 8](#_Toc107238586)

[8.2.2 Per Protocol Population (PPP) 8](#_Toc107238587)

[8.2.3 Safety Population 8](#_Toc107238588)

[8.3 Subgroup analysis 8](#_Toc107238589)

[8.4 Missing Data 8](#_Toc107238590)

[9 Summary of Study Data 8](#_Toc107238591)

[9.1 Subject Disposition 9](#_Toc107238592)

[9.2 Protocol Deviations 10](#_Toc107238593)

[9.3 Demographic and Baseline Variables 10](#_Toc107238594)

[9.4 Concurrent Illnesses and Medical Conditions 10](#_Toc107238595)

[9.5 Treatment Compliance 10](#_Toc107238596)

[10 Efficacy Analyses 10](#_Toc107238597)

[10.1 Primary Efficacy Analysis 11](#_Toc107238598)

[10.2 Secondary Efficacy Analyses 11](#_Toc107238599)

[10.3 Exploratory Efficacy Analyses 11](#_Toc107238600)

[11 Safety Analyses 11](#_Toc107238601)

[11.1 Adverse Events 11](#_Toc107238602)

[11.2 Clinical Laboratory Evaluations 12](#_Toc107238603)

[11.3 Vital Sign Measurements 12](#_Toc107238604)

[11.4 Physical Examination and Pregnancies 12](#_Toc107238605)

[12 Reporting Conventions 13](#_Toc107238606)

[13 References 13](#_Toc107238607)

[14 Listing of Tables, Listings and Figures 13](#_Toc107238608)

# Abbreviations and Definitions

| AE | Adverse Event |
| --- | --- |
| CRF | Case Report Form |
| IMP | Investigational Medical Product |
| SAP | Statistical Analysis Plan |
| RCT | randomized control rail |
| rTMS | repetitive transcranial magnetic stimulation |
| M1 | primary motor cortex |
| ICIQ-UI SF | International Consultation on Incontinence Questionnaire Urinary Incontinence Short Form |
| OABSS | Overactive Bladder Symptom Score |
| sEMG | surface electromyography |
| PFM | pelvic floor muscles |
| PSI | post-stroke urinary incontinence |
| LF-rTMS | low-frequency rTMS |
| FAP | Full Analysis Population |
| PPP | Per Protocol Population |

#

# Introduction

## Preface

This statistical analysis plan (SAP) describes the analyses and data presentations for Wei Jiang’s study protocol “Effectiveness of repetitive transcranial magnetic stimulation against post-stroke urinary incontinence: a study protocol for a randomized controlled trial”. It contains definitions of analysis populations, derived variables, and statistical methods for the analysis of efficacy and safety.

These analyses include one final analysis. Throughout this SAP, the treatment arms will be referred to as “low-frequency rTMS” for post-stroke urinary incontinence and the control arm will be "sham rTMS “for post-stroke urinary incontinence”. The purpose of the SAP is to ensure the credibility of the study findings by specifying the statistical approaches to the analysis of study data prior to database lock for the final analysis. All statistical analyses detailed in this SAP will be conducted using SPSS 19.0 (SPSS, Inc, Chicago, IL), or higher.

## Scope of the analyses

These analyses will assess the efficacy and safety of contolesional M1 area [LF-rTMS] on PSI in comparison with the [Sham rMTS] and will be included in the clinical study report.

# Study Objectives

## Study Objectives

The primary objective of the study is:

• to compare the efficacy of LF-rTMS versus sham rTMS on bladder capacity in subjects with post-stroke urinary incontinence

The bladder capacity came from urodynamic testing. During that test, we could also acquire other parameters. For example, detrusor contraction, bladder compliance, the sensation of bladder filling, any leakage, bladder capacity, detrusor pressure at maximum flow, maximum flow rate, and postvoid residual urine volume.

The secondary objectives of the study are:

• to compare the safety of LF-rMTS versus sham rTMS(no formal testing will be provided)

• to compare the efficacy of LF-rMTS versus sham rTMS using other parameters of efficacy:

− The self-reporting questionnaire of ICIQ-UI SF and OABSS.

− Surface EMG of the pelvic floor muscles (PFM) activities.

# Study Methods

## General Study Design and Plan

This is a single-center, randomised double-blind, placebo-controlled superiority trial. Patients will be randomized 1:1 to receive either LF-rTMS or sham rTMS therapy.

The recruitment stops when there are 70 participants included in each group.

- rTMS group: 70, who will accept contralesional M1 LF-rTMS once a day, 5days/week for continues 4weeks.
- sham rTMS group: 70, who will accept contralesional M1 sham rTMS once a day, 5days/week for continues 4weeks.

## Inclusion-Exclusion Criteria and General Study Population

**The inclusion criteria are as follows:**

(1) onset of stroke between 1 to 12 months

(2) suffered from urinary incontinence after stroke

(3) aged over 30 years old of any gender

(4) no special medications administered for urinary incontinence

(5) the ability to understand and agree to the trial procedures and to sign an informed consent form in accordance with the national legislation.

**The exclusion criteria are:**

(1) any urinary dysfunction before stroke

(2) bilateral lesion in the brain

(3) urinary tract infection

(4) the presence of an unstable medical condition or an uncontrolled known systemic disease

(5) contraindications to rTMS (seizure, cardiac pacemaker, or ears nest implants)

(6) refuse to continue of the intervention.

## Randomization and Blinding

- Participants will be randomly allocated to receive low frequency (LF) rTMS over the contralesional primary motor cortex(M1) or sham rTMS. The randomized number and sequence will be generated through SPSS Version 19.0 by an independent statistician, who will not take part in assessments and execution in this trial. An outsider research-assistant will allocate these numbers to participants according to the order of inclusion. After that the therapist and all eligible participants will be informed of the results of group allocation by an independent clinician via short message.
- As the physicians and the therapists administering the rTMS protocol, they cannot be blinded. However, both the researchers (outcome assessors and statistical analysts) and participants are blinded to the group assignment and the blindness will never be broken prior to the completion of study unless adverse events happen. Meanwhile, the randomized sequence of allocation will be replaced by some unrelated codes, such as I and II. In this study, code I means rTMS group and code II means sham rTMS group. Each parameter of randomization will be kept in a special sealed opaque envelope as previously reported.

## Study Assessments

Schedule of enrollment, interventions, and assessments.


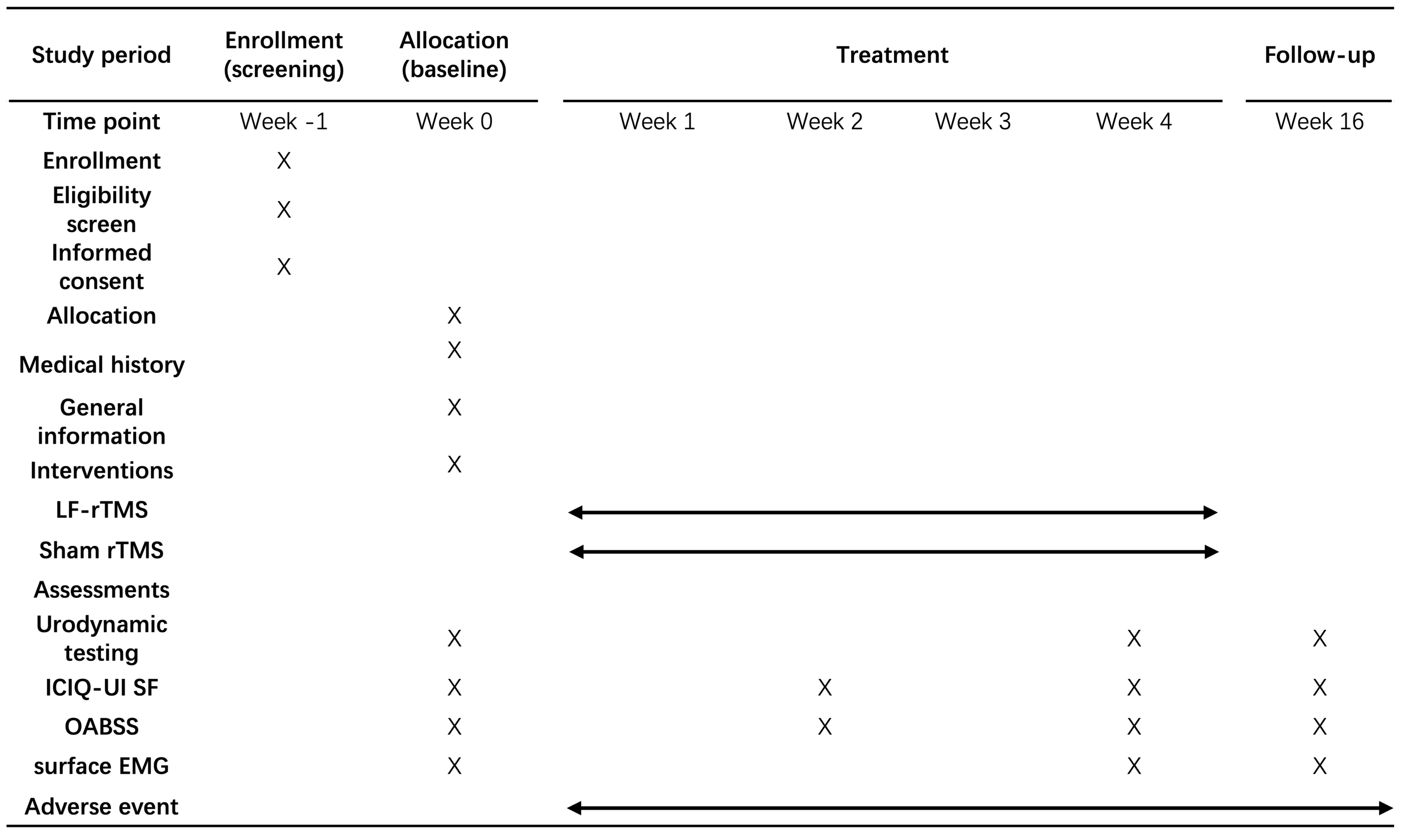


- Identification of any number ranges for numeric endpoints along with their corresponding text descriptors.
  - Items are measured on a 0-21 International Consultation on Incontinence Questionnaire Urinary Incontinence Short Form (ICIQ-UI SF) for which 0–7 points for mild UI, 8–12 points for moderate UI and 13–21 points for severe urinary incontinence.
  - Items are measured on a 0-15 Overactive Bladder Symptom Score (OABSS) for which severity depending on the OABSS total score. it is defined as mild ≤5, moderate 6-11, severe ≥12.

# Sample Size

We aim to investigate the efficacy of LF rTMS for improving bladder function in PSI individuals. However, the primary outcomes for most previous studies are the pad test and urinary continence questionnaire, including the study previously referenced.

In this trial, we chose bladder capacity as one of our primary outcomes. Based on our previous observation (unpublished data), the mean and SD of bladder capacity in PSI patients were approximately 300 and 100 ml, respectively, and a bladder capacity increase of more than 50 ml was thought to be a therapeutic effect42. According to those results, a sample size of 63 participants in each group was calculated to sufficiently detect the target effect size (0.5) with a type I error of 5% (α = 0.05) and 80% power (β = 0.20) by Gpower V.3.1.9.2 software. We will add 10% to 25% more participants to account for potential loss to follow-up, resulting in a final enrolment goal of 140 participants (70 per group).

# General Analysis Considerations

## Timing of Analyses

The final analysis will be performed when all the subjects have completed the follow-up visit and all data will be transferred to the electronic edition, having been documented as meeting the cleaning and approval requirements of the Data Monitoring Committee and after the finalization and approval of this SAP document.

## Analysis Populations

The primary efficacy analyses will be performed on the Intent-to-treat (ITT) population for the primary and secondary endpoints. Primary and secondary endpoints will also be analyzed for the modified Intent-to-treat (mITT) population. Sensitivity analyses will be conducted for the primary and secondary endpoints based on the ITT population. All ITT and mITT analyses will be based on randomized treatment groups.

- - 1. **Full Analysis Population (FAP) (Intention-to-treat Population or Modified ITT)**
- All subjects who were randomized, regardless of whether they received study treatment or not.
- The mITT population is defined as all randomized subjects who have received at least 10 times of rTMS therapy and at least one post-baseline assessment for efficacy.
- The efficacy analysis will also be performed on the mITT population as supportive evidence and/or sensitivity analysis. Subjects will be analyzed according to the treatment arm to which they are initially assigned.
- FAP will be the primary set that being analyzed.

### Per Protocol Population (PPP)

- *All subjects who did not substantially deviate from the protocol as to be determined on a per-subject basis at the trial steering committee immediately before database lock.*
- *PPP* will be the secondary set that being analyzed.

### Safety Population

- The safety population is defined as all subjects who have received at least one time of study rTMS therapy.
- The safety population will be used for all safety analyses. Subjects will be analyzed according to the treatment they initially received.

## Subgroup analysis

In addition to analyses that include the ITT population, subgroup analyses may also be performed on other important factors such as demographic and baseline characteristics as needed:

- Age (< 65; ≥ 65 years)
- Sex (male; female)
- type of stroke course of stroke(<6 ; ≥6 months)
- bladder capacity before treatment (<300 ; ≥300 millilitres)

## Missing Data

Last Observation Carried Forward (LOCF) will be employed to the analysis of longitudinal repeated measures data where some follow-up observations may be missing.

In a LOCF analysis, a missing follow-up visit value is replaced by (imputed as) that subject’s previously observed value, i.e. the last observation is carried forward.

# Summary of Study Data

All continuous variables will be summarized using the following descriptive statistics: n (non-missing sample size), mean, standard deviation, median, maximum and minimum. The frequency and percentages (based on the non-missing sample size) of observed levels will be reported for all categorical measures. In general, all data will be listed, sorted by site, treatment and subject, and when appropriate by visit number within subject. All summary tables will be structured with a column for each treatment in the order (sham rTMS, LF-rTMS ) and will be annotated with the total population size relevant to that table/treatment, including any missing observations.

It may be worthwhile to document that the sample size of non-missing values for univariable summary statistics may be larger than the sample size of non-missing values in a complete-case analysis used in a typical primary regression analysis, as different patients may have missing values in different variables, such as baseline covariates and the endpoints. Generally, we would not want to exclude observations by repeating the summary statistics in a “complete case population.” Exclusions may imply the excluded observations are systematically different, thus contradicting a typical Missing At Random assumption, and could also be considered as a post-hoc subgroup analysis.

## Subject Disposition

Subject disposition will be tabulated using frequency and percent for all enrolled subjects. Subject disposition includes the number of subjects in the following populations by treatment arm: ITT, mITT, and safety populations.

Primary reasons for discontinuation from each study treatment will be collected on the CRF and will be listed in subject data listings as well as summarized, using frequency and percent, for all randomized subjects including the following categories:

- Adverse event(s)
- Withdrawal of consent
- Death
- Lost to follow-up
- Protocol violation
- Other

Number of subjects who are ongoing in treatment, number of subjects who are ongoing in follow-up, and number of subjects who completed treatment will also be tabulated.

Number and percent of subjects who entered the follow-up phase with or without progression will be summarized. Primary reasons for study discontinuation will be collected on the CRF and will be listed in subject data listings as well as summarized, using frequency and percent, for all randomized subjects including the following categories:

- Withdrawal of consent
- Death
- Lost to follow up
- Protocol violation

Duration of study participation, defined as from the date of randomization to the date of discontinuation of the study (or the date of the last visit), will be summarized.

Subject listings will be provided for randomized subjects, subjects discontinued from treatment, subjects discontinued from the study, and subjects who are excluded for a reason. The screen failures will be tabulated by unmet inclusion/exclusion criteria. A summary tabulation will be provided for subjects enrolled by study center.

## Protocol Deviations

Protocol deviations/violations are identified and assessed by the clinical research physician or designee following company standard operational procedure. The protocol violations will be summarized by treatment arm in the ITT population.

Protocol deviations will be summarized using frequency tabulations and listed under individual subject listings with no derivation as this information is from the clinical trial monitoring system.

A by-subject listing of subjects with protocol violations and deviations in the ITT population will be provided.

## Demographic and Baseline Variables

Subjects’ age, height, weight, and other baseline characteristics (e.g., type of stroke, location of the lesion) will be summarized using descriptive statistics, while sex, and other categorical variables (e.g., baseline urodynamic testing results, the severity of the urine incontinence), will be provided using frequency tabulations.

The following items will be summarized by treatment arm.

- Bladder capacity
- Detrusor pressure
- Residual volume
- Maximum flow rate
- Score of ICIQ-UI SF
- OABSS
- pelvic floor muscle (PFM) activities(5-s maximum voluntary contractions)

## Concurrent Illnesses and Medical Conditions

All medical history will be coded using the Medical Dictionary for Regulatory Activities (MedDRA®) Version 20.0 or later. The version of the MedDRA will be indicated in the footnote of relevant tables based on the current version used in the clinical data. A summary of the medical and surgical history will be presented by MedDRA system organ class (SOC) and preferred term (PT) in descending order of frequency by SOC, and then within each SOC by descending order of PT by the LF-rTMS group. By-subject listings will display medical history including all relevant data collected on the CRF.

## Treatment Compliance

The table of tables document may be given as a reference to provide the finest level of detail needed to produce the report and to avoid duplication. The therapy records will be employed to measure treatment compliance. Those who accomplished over 20 times rTMS will be recognized as good compliance. The summary statistics will be produced in accordance with section 9.

# Efficacy Analyses

All efficacy analyses will be performed on the ITT population. Key efficacy analyses will be performed on the mITT population as supportive evidence and to assess the robustness of efficacy findings. Subjects will be analyzed according to randomized groups. Data will be summarized by treatment group. N, Mean, Standard Deviation, Minimum and Maximum will summarize continuous efficacy variables, whereas number and percent will summarize categorical efficacy variables.

All stratified analyses will be based on CRF data.

First, the comparison of the baseline data between the two groups will be performed using Student’s t test (e.g. bladder capacity), chi-squared test (e.g. type of stroke), or nonparametric test according to the type and distribution of variables. To verify the therapeutic efficacy of our intervention, comparisons of all the outcomes that are categorized as continuous variables and shown in mean scores will be included in the analysis by independent sample t-test (e.g. bladder capacity) or non-parametric test (Wilcoxon and Mann–Whitney U tests) depending on the distribution of the data. If the data are distributed normally and the variances are equal, independent samples t-tests will be used for comparisons of the before and after treatment changes between groups, including the mean differences between groups, with 95% CIs.

Treatment groups will be tested at the 2-sided 5% significance level.

## Primary Efficacy Analysis

The primary analysis will be performed to compare the efficacy of LF-rTMS versus sham rTMS on bladder capacity in subjects with post-stroke urinary incontinence. The experimental arm will be declared superior if the double-sided p-value from the t-test is < 0.05.

Our hypothesis is that real rTMS stimulation will elicit greater changes in balder capacity than sham rTMS stimulation. Therefore, the main analysis is the comparison of changes between groups, measured pre- and post-intervention. If significant differences are found before and after treatment changes between groups, we may conclude the efficacy of rTMS to participants with PSI.

Other parameters, such as detrusor contraction, bladder compliance, the sensation of bladder filling, any leakage, bladder capacity, detrusor pressure at maximum flow, maximum flow rate, and postvoid residual urine volume will also be considered in the comparison.

## Secondary Efficacy Analyses

The secondary efficacy analyses will be performed to compare the efficacy of LF-rTMS versus sham rTMS on ICIQ-UI SF, OABSS and surface EMG of the pelvic floor muscles (PFM) activities in subjects with post-stroke urinary incontinence. The experimental arm will be declared superior if the double-sided p-value from the t-test is < 0.05 in favour of the experimental (LF-rTMS) arm.

## Exploratory Efficacy Analyses

The summary statistics will be produced in accordance with section 8.

# Safety Analyses

All subjects who receive at least one dose of study treatment(rTMS) will be included in the safety analyses.

## Adverse Events

AEs will be graded according to the criteria in this table, which is from the NCI CTCAE version 4.03 Table for Grading the Severity of Adult Adverse Events.

| Grade |  | Description |
| --- | --- | --- |
| Grade 1 (mild) |  | Asymptomatic or mild symptoms; clinical or diagnostic  observations only; intervention not indicated |
| Grade 2 (moderate) |  | Minimal, local or non-invasive intervention indicated; limiting age-appropriate instrumental ADL |
| Grade 3 (severe) |  | Medically significant but not immediately life-threatening; hospitalization or prolongation of hospitalization indicated; disabling; limiting self-care ADL |
| Grade 4 (life-threatening) |  | Life-threatening consequences; urgent intervention  indicated |
| Grade 5 (death) |  | Death related to AE |

ADL, activities of daily living; AE, adverse event; CTCAE, Common Terminology Criteria for Adverse Events; NCI, National Cancer Institute.

When calculating the incidence of adverse events, or any sub-classification thereof by treatment, time period, severity, etc., each subject will only be counted once and any repetitions of adverse events will be ignored; the denominator will be the total population size.

A treatment-emergent adverse events (TEAEs) are is defined as any AE occurring or worsening on or after the first rTMS treatment of the study and after the last treatment of the study

If a subject experienced the same rTMS multiple times then the subject will be counted only once within the particular treatment and by greatest severity. The following TEAEs will be summarized by rTMS and Sham for each group: TEAEs leading to study treatment discontinuation, TEAEs leading to rTMS intensity reduction, TEAEs related to study medication, severe TEAEs, serious TEAEs related to study treatment, serious TEAEs leading to study medication discontinuation, NCI-CTCAE Grade 3 or and 4 TEAEs, Grade 3 and 4 TEAEs leading to study treatment discontinuation, Grade 5 TEAEs and AEs of particular interest.

Adverse events of special interest include:

- epilepsy
- fatigue
- dizziness
- headache

## Clinical Laboratory Evaluations

Clinical laboratory values will be graded according to NCI CTCAE Version 4.0 for applicable tests. Shift from baseline to worst severity grade observed during the treatment will be displayed by treatment. Normal ranges will be used to determine the categories if High, Low, and Normal for lab tests that have no severity grade.

Listings of clinical laboratory data with abnormal flags will be provided by subject and test.

## Vital Sign Measurements

Vital signs will be presented in a listing, within and above the normal ranges, will be displayed in the listing for each treatment. Normal ranges are defined as follows:

- Systolic BP Normal (90 through 119 mmHg, inclusive)
- Diastolic BP Normal (60 through 79 mmHg, inclusive)
- Body Temperature Normal (36.1 through 37.8°C, inclusive)
- Pulse Normal (60 through 100 bpm, inclusive)

## Physical Examination and Pregnancies

The physical examinations will be summarized by each visit.

Pregnancy testing results for female subjects of childbearing potential will be summarized by visit.

# Reporting Conventions

P-values ≥0.001 will be reported to 3 decimal places; p-values less than 0.001 will be reported as “<0.001”. The mean, standard deviation, and any other statistics other than quantiles, will be reported to one decimal place greater than the original data. Quantiles, such as median, or minimum and maximum will use the same number of decimal places as the original data. Estimated parameters, not on the same scale as raw observations (e.g. regression coefficients) will be reported to 3 significant figures.

# References

White IR and Thompson SG (2005). Adjusting for partially missing baseline measurements in randomized trials. Statistics in Medicine, 24, 993-1007.

US Department of Health and Human Services. Common terminology criteria for adverse events (CTCAE) version 4.03. 2010[J]. USA: National Institutes of Health, National Cancer Institute, 2016.

Wang S, Lv J, Feng X, et al. Efficacy of electrical pudendal nerve stimulation versus transvaginal electrical stimulation in treating female idiopathic urgency urinary incontinence[J]. The Journal of Urology, 2017, 197(6): 1496-1501.

Chan A W, Tetzlaff J M, Gøtzsche P C, et al. SPIRIT 2013 explanation and elaboration: guidance for protocols of clinical trials[J]. Bmj, 2013, 346.

# Listing of Tables, Listings and Figures

Table 1. Clinical characteristics of the subjects in both groups.

|  | rTMS group (*n*=) | sham group (*n*=) | *p*-value |
| --- | --- | --- | --- |
| Age |  |  |  |
| Sex, *n* (%) |  |  |  |
| Male |  |  |  |
| Female |  |  |  |
| Stroke type, *n* (%) |  |  |  |
| Infarction |  |  |  |
| Hemorrhage |  |  |  |
| Lesion side |  |  |  |
| Left |  |  |  |
| Right |  |  |  |
| HAMD |  |  |  |
| Stroke onset,  month |  |  |  |

Table 2. Trial results for the primary and secondary outcomes

|  |  | **Baseline** | | 2W | | 4W | | Follow-up | |
| --- | --- | --- | --- | --- | --- | --- | --- | --- | --- |
|  |  | rTMS group | Sham  group | rTMS group | Sham  group | rTMS group | Sham  group | rTMS group | Sham  group |
| Primary outcome | Bladder capacity |  |  |  |  |  |  |  |  |
|  | Detrusor pressure |  |  |  |  |  |  |  |  |
|  | Residual volume |  |  |  |  |  |  |  |  |
|  | Maximum flow rate |  |  |  |  |  |  |  |  |
| Secondary outcome | ICIQ-UI SF |  |  |  |  |  |  |  |  |
|  | OABSS |  |  |  |  |  |  |  |  |
|  | surface EMG |  |  |  |  |  |  |  |  |

Table 3. Safety outcomes in both groups

|  |  | rTMS group | Sham group |
| --- | --- | --- | --- |
| Randomize | N(%) |  |  |
| Accompaniment | N(%) |  |  |
| Not meeting the inclusion criteria | N(%) |  |  |
| Protocol violation | N(%) |  |  |
| Withdrawal of consent | N(%) |  |  |
| Adverse event(s) | N(%) |  |  |
| Lost to follow-up | N(%) |  |  |
| Recurrence of stroke | N(%) |  |  |
| Death | N(%) |  |  |
| Safety analysis set | N(%) |  |  |
| Efficacy analysis set | N(%) |  |  |
| FAP | N(%) |  |  |
| PPP | N(%) |  |  |

FAP: Full Analysis Population; PPP: Per Protocol Population.
